# Mutations in *GMPPB* cause congenital myasthenic syndrome and bridge myasthenic disorders with dystroglycanopathies

**DOI:** 10.1093/brain/awv185

**Published:** 2015-07-01

**Authors:** Katsiaryna Belaya, Pedro M. Rodríguez Cruz, Wei Wei Liu, Susan Maxwell, Simon McGowan, Maria E. Farrugia, Richard Petty, Timothy J. Walls, Maryam Sedghi, Keivan Basiri, Wyatt W. Yue, Anna Sarkozy, Marta Bertoli, Matthew Pitt, Robin Kennett, Andrew Schaefer, Kate Bushby, Matt Parton, Hanns Lochmüller, Jacqueline Palace, Francesco Muntoni, David Beeson

**Affiliations:** 1 Neurosciences Group, Nuffield Department of Clinical Neurosciences, Weatherall Institute of Molecular Medicine, University of Oxford, Oxford, OX3 9DS, UK; 2 Nuffield Department of Clinical Neurosciences, John Radcliffe Hospital, Oxford, OX3 9DU, UK; 3 Computational Biology Research Group, Weatherall Institute of Molecular Medicine, University of Oxford, Oxford, OX3 9DS, UK; 4 Department of Neurology, Institute of Neurological Sciences, Southern General Hospital, Glasgow, UK; 5 Department of Neurology, Royal Victoria Infirmary, Newcastle upon Tyne, NE1 4LP, UK; 6 Medical Genetics Laboratory, Alzahra University Hospital, Isfahan University of Medical Sciences, Isfahan, Iran; 7 Neurology Department, Neuroscience Research Centre, Isfahan University of Medical Sciences, Isfahan, Iran; 8 Structural Genomics Consortium, University of Oxford, Oxford, OX3 7DQ, UK; 9 Institute of Genetic Medicine, John Walton Muscular Dystrophy Research Centre, MRC Centre for Neuromuscular Diseases, Newcastle University, Newcastle upon Tyne, NE1 3BZ, UK; 10 MRC Centre for Neuromuscular Diseases, UCL Institute of Neurology and National Hospital for Neurology and Neurosurgery, Queen Square, London, UK; 11 Department of Clinical Neurophysiology, Great Ormond Street Hospital for children NHS foundation trust, London WC1N 3JH; 12 Dubowitz Neuromuscular Centre and MRC Centre for Neuromuscular Diseases, UCL Institute of Child Health, London, WC1N 1EH, UK

**Keywords:** congenital myasthenic syndrome, glycosylation, GMPPB, neurotransmission defect, dystroglycan

## Abstract

Congenital myasthenic syndromes are inherited disorders that arise from impaired signal transmission at the neuromuscular junction. Mutations in at least 20 genes are known to lead to the onset of these conditions. Four of these, *ALG2*, *ALG14*, *DPAGT1* and *GFPT1*, are involved in glycosylation. Here we identify a fifth glycosylation gene, *GMPPB*, where mutations cause congenital myasthenic syndrome. First, we identified recessive mutations in seven cases from five kinships defined as congenital myasthenic syndrome using decrement of compound muscle action potentials on repetitive nerve stimulation on electromyography. The mutations were present through the length of the *GMPPB*, and segregation, *in silico* analysis, exon trapping, cell transfection followed by western blots and immunostaining were used to determine pathogenicity. GMPPB congenital myasthenic syndrome cases show clinical features characteristic of congenital myasthenic syndrome subtypes that are due to defective glycosylation, with variable weakness of proximal limb muscle groups while facial and eye muscles are largely spared. However, patients with GMPPB congenital myasthenic syndrome had more prominent myopathic features that were detectable on muscle biopsies, electromyography, muscle magnetic resonance imaging, and through elevated serum creatine kinase levels. Mutations in GMPPB have recently been reported to lead to the onset of muscular dystrophy dystroglycanopathy. Analysis of four additional GMPPB-associated muscular dystrophy dystroglycanopathy cases by electromyography found that a defective neuromuscular junction component is not always present. Thus, we find mutations in *GMPPB* can lead to a wide spectrum of clinical features where deficit in neuromuscular transmission is the major component in a subset of cases. Clinical recognition of *GMPPB*-associated congenital myasthenic syndrome may be complicated by the presence of myopathic features, but correct diagnosis is important because affected individuals can respond to appropriate treatments.

## Abstract (precis)

Congenital myasthenic syndromes are associated with impairments in neuromuscular transmission. Belaya *et al.* show that mutations of the glycosylation pathway enzyme GMPPB, which has previously been implicated in muscular dystrophy dystroglycanopathy, also cause a congenital myasthenic syndrome. This differential diagnosis is important to ensure that affected individuals receive appropriate medication.

## Graphical abstract

**Figure:**
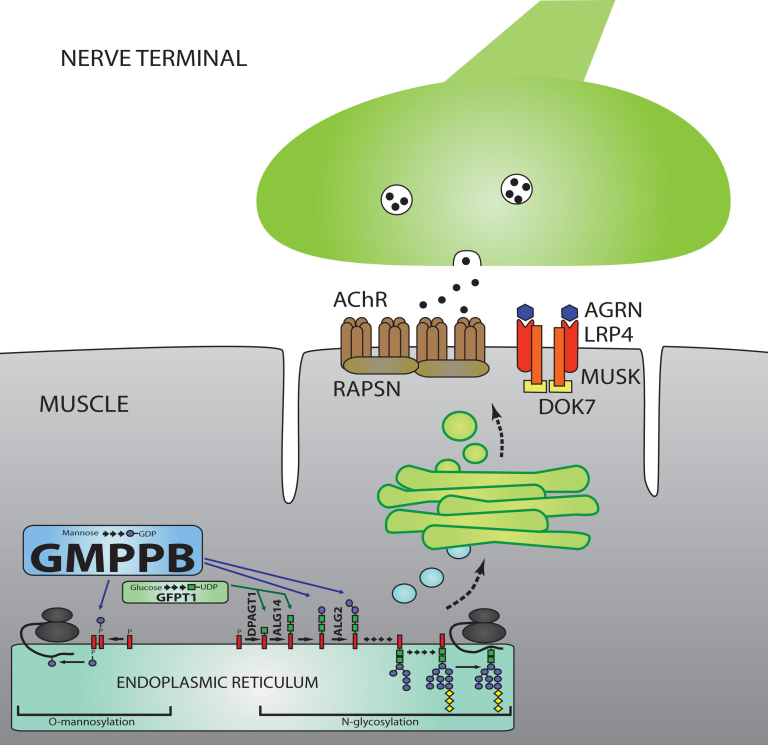
Congenital myasthenic syndromes are associated with impairments in neuromuscular transmission. Belaya *et al.* show that mutations of the glycosylation pathway enzyme GMPPB, which has previously been implicated in muscular dystrophy dystroglycanopathy, also cause a congenital myasthenic syndrome. This differential diagnosis is important to ensure that affected individuals receive appropriate medication.

## Introduction

**Figure 1:**
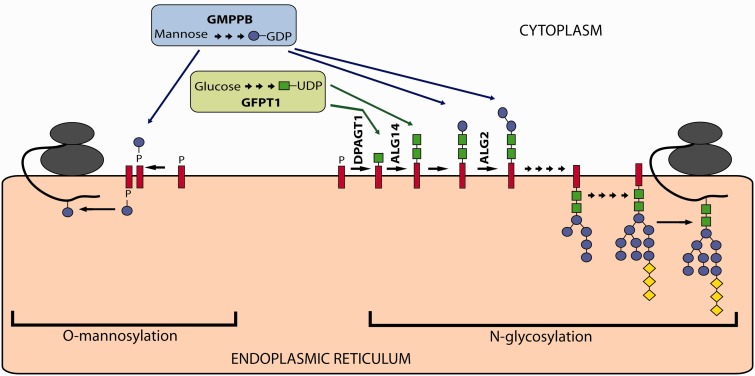
**Simplified scheme of *N*-linked and *O*-linked glycosylation.** The scheme shows five glycosylation genes associated with CMS.

Congenital myasthenic syndromes (CMS) are a rare and heterogeneous group of inherited disorders caused by mutations in genes encoding proteins that are essential for neuromuscular transmission. All CMS share the clinical feature of fatigable weakness, but age at onset, presenting symptoms, distribution of weakness, and response to treatment differ depending on the molecular mechanism that results from the genetic defect (Engel, 2012; Cruz *et al.*, 2014). In most cases impaired transmission can be detected by EMG, and a decrement in the amplitude of compound muscle action potentials in response to repetitive nerve stimulation and/or increased jitter represent crucial diagnostic criteria for these disorders. Mutations in at least 20 different genes are known to lead to the development of CMS (Cruz *et al.*, 2014). Four of these, *ALG2*, *ALG14*, *DPAGT1* and *GFPT1*, are recently identified genes encoding proteins involved in glycosylation (Fig. 1) (Senderek *et al.*, 2011; Belaya *et al.*, 2012; Cossins *et al.*, 2013). *ALG2*, *ALG14* and *DPAGT1* are involved specifically in *N*-linked protein glycosylation. *GFPT1* is involved in the synthesis of UDP-GlcNAc, a nucleotide sugar used as a building block by several glycosylation pathways, including *N*- and *O*-linked glycosylation. These four subtypes of glycosylation-CMS have characteristic clinical features that help to distinguish them from other CMS subtypes. The most commonly affected muscles are proximal limb muscles, with limited ocular or facial involvement. Identified cases may respond to treatment with pyridostigmine or a combination of pyridostigmine and salbutamol. The pathogenic mechanism of these disorders is not entirely understood, but it has been proposed that one contributing pathogenic molecular mechanism underlying the disorders is due to the abnormal glycosylation of acetylcholine receptor (AChR) subunits (Belaya *et al.*, 2012; Cossins *et al.*, 2013; Zoltowska *et al.*, 2013). Glycosylation of AChRs is required for assembly of AChR pentamers and for efficient export of the receptors to the cell surface (Nomoto *et al.*, 1986; Gehle and Sumikawa, 1991; Gehle *et al.*, 1997; Wanamaker *et al.*, 2003). Disruption of glycosylation leads to a lack of AChRs at the endplate region, and a reduced synaptic response to acetylcholine. Other neuromuscular junction proteins are likely to be affected by abnormal glycosylation and might further contribute to the phenotype (Selcen *et al.*, 2014).

There remain a number of CMS cases that have a clear neuromuscular transmission defect, but no identified mutation in the known CMS-associated genes. Here, we use whole exome sequencing to identify CMS-causing mutations in GDP-mannose pyrophosphorylase B (*GMPPB*). Mutations in *GMPPB* have been previously associated with the development of a muscular dystrophy dystroglycanopathy (MDDG), which is caused by the defective *O*-glycosylation of α-dystroglycan (Carss *et al.*, 2013). GMPPB-MDDG patients present with a characteristic set of dystrophic features, which are often accompanied by a variable degree of structural brain and eye abnormalities. We identify a spectrum of clinical phenotypes associated with *GMPPB* mutations that stretched from defective neuromuscular transmission forming the major symptomatic component to GMPPB-MDDG cases in which there is no detectable neuromuscular junction defect.

## Materials and methods

### Patient data

Patient consent for use of data was obtained with ethical approval OXREC B: 04.OXB.017 and Oxfordshire REC C 09/H0606/74. Patient neurophysiology, muscle MRI, and serum creatine kinase levels were measured by standard clinical techniques.

### Whole exome sequencing

Patient genomic DNA was extracted from peripheral blood using Nucleon® kit (Gen-Probe Life Sciences Ltd). Exome library was captured from 3 µg of genomic DNA using Agilent SureSelect XT Human All Exon v.4 kit. The libraries were sequenced by 100 nt paired-end reads on Illumina HiSeq platform. The obtained sequences were mapped to human genome build hg19 by using Novoalign software (Novocraft Technologies). Variants were called using Samtools program (Li *et al.*, 2009). Variants were filtered out if their population frequency was ≥0.01 according to 1000 Genomes Project (European subset) (Genomes Project *et al.*, 2010). We used ANNOVAR software to annotate and separate non-synonymous substitutions, indel variants and splicing mutations (Wang *et al.*, 2010). As CMS are usually inherited in an autosomal recessive manner, we focused on all genes that have either one or more homozygous variant, or two or more heterozygous variants in the same gene. We further filtered the obtained variants against an in-house database of 14 exomes from cases with unrelated disorders, and manually removed misaligned or low quality reads.

### Analysis of GMPPB expression and localization

IMAGE clone containing cDNA of *GMPPB* was purchased from Source Bioscience Lifesciences (IRAUp969D1013D). *GMPPB* coding sequence was subcloned into the mammalian expression vector pcDNA3.1-hygro (Invitrogen). Mutations were introduced using site-directed mutagenesis using QuikChange® kit from Stratagene. All sequences were confirmed by Sanger sequencing. HEK293 cells were transfected with wild-type or mutant constructs. Forty-eight hours after transfection, the cells were harvested and lysed by rotating in cold lysis buffer [10 mM Tris (pH 7.5), 100 mM NaCl, 1 mM EDTA, 1% Triton™ X-100, mammalian protease inhibitor cocktail form Sigma] for 1 h. Cell extracts were centrifuged and resuspended into protein loading buffer. Protein extracts were separated by sodium dodecyl sulphate polyacrylamide gel electrophoresis and transferred onto polyvinylidene difluoride membrane. The membrane was incubated with primary anti-GMPPB antibody (Abcam ab154061) and secondary anti-rabbit antibody conjugated to horseradish peroxidase (Dako). Detection was performed by using ECL (GE Healthcare).

For the analysis of GMPPB localization, C2C12 muscle cells were transfected with wild-type or mutant GMPPB constructs using the Neon® Transfection System (Life Technologies). Transfection was performed according to manufacturer’s recommendations using 2 µg of each DNA construct. The cells were fixed 24 h after electroporation. Fixed cells were permeabilized using 0.1% Triton in phosphate-buffered saline, blocked in 1% bovine serum albumin, stained with primary anti-GMPPB antibody and secondary anti-rabbit antibody conjugated to Alexa Fluor® 488 (Invitrogen). Imaging was performed on the inverted Zeiss LSM 510 META confocal microscope.

#### Exon trapping

We cloned *GMPPB* exons 2, 3, 4 and flanking intronic sequences into the pET01 vector (MoBiTec). The c.130-3C>G mutation was introduced by site-directed mutagenesis using QuikChange® kit from Stratagene and confirmed by Sanger sequencing. Control and mutant vector DNA were electroporated into the human rhabdomyosarcoma cell line TE671 using the Neon® electroporator (Invitrogen). Total RNA was purified 48 h after transfection, reverse transcribed into cDNA using RETROscript® kit (Ambion). Complementary DNA was amplified using primers specific to the vector exons. The amplicons were run on agarose/TBE (Tris-borate-EDTA) gels, visualized under UV/ethidium bromide and then gel purified and sequenced.

## Results

### Clinical features

#### Cases 1–7

**Figure 2:**
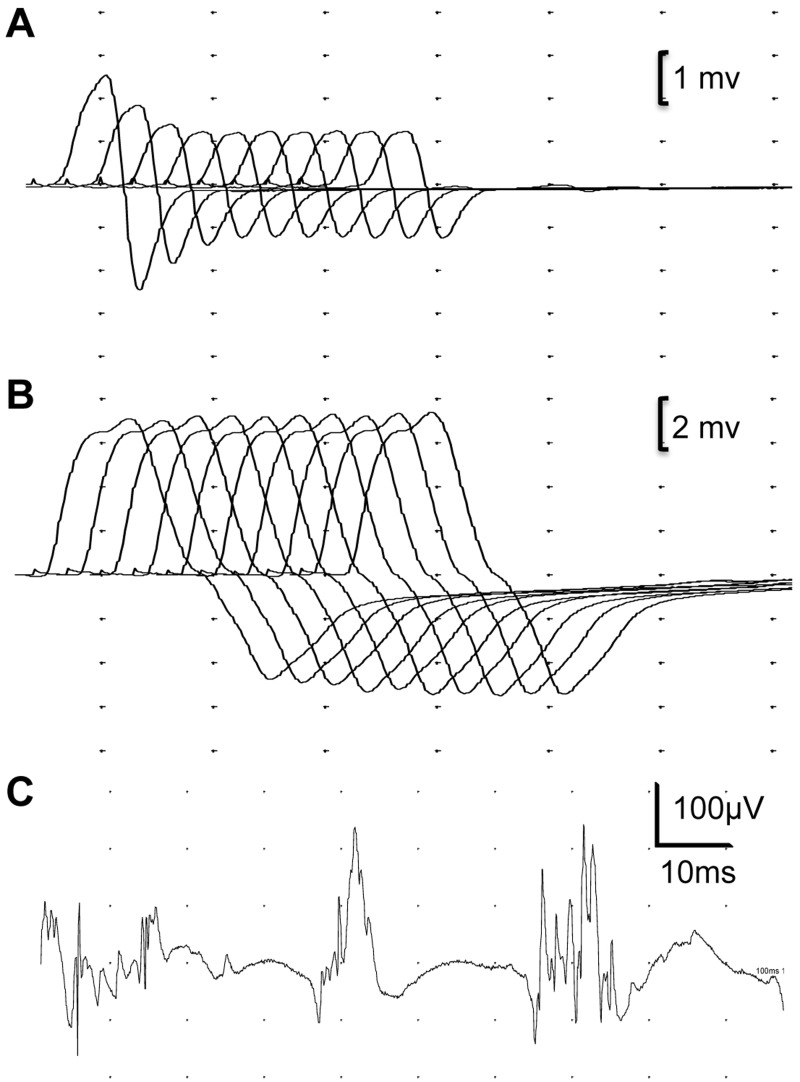
**Neurophysiological examination of Case 3 using repetitive nerve stimulation and concentric needle EMG.** Repetitive nerve stimulation studies performed on right anconeus muscle showed 50% amplitude decrement (**A**) in compound muscle action potentials while there was no change in compound muscle action potential amplitude with repetitive stimulation of right abductor digiti minimi muscle (**B**). Concentric needle EMG examination of the right biceps muscle at low force of contraction showed low amplitude, polyphasic motor unit action potentials.

Cases 1–7 demonstrate the hallmarks of myasthenic disorders with fluctuating fatigable muscle weakness and clear decrement of compound muscle action potential on EMG during repetitive nerve stimulation (Table 1 and Fig. 2). Cases 1, 2, 3 and 7 are unrelated individuals of Caucasian descent. Cases 4, 5 and 6 are siblings from a consanguineous marriage of first cousins once removed (of Iranian origin). Typically, presentation was in adolescence or adulthood and included limited march tolerance, inability to run and lift weights, and difficulties in rising from the floor. In retrospect the onset of muscle weakness may be earlier because the majority of cases recall being slower than their peers at school, not excelling in sports, and some delay of motor milestones. Muscle weakness was found to be most prominent in proximal limb muscles, while facial, eye, and bulbar muscles are largely spared. Ptosis is only present in Case 4. The patients reported activity-dependent fatigability and improvement with rest. In addition, they also presented fluctuations of muscle strength over time, from days to weeks or even months. On occasions, deteriorations have followed recognizable precipitants such as viral infections or menstruation but they have also occurred spontaneously. As an example, Case 1 had fluctuations of muscle strength over the course of weeks. At best she is able to walk short distances (∼20 m) using a walking aid. At worst, she cannot walk at all, is wheelchair bound and needs assistance to stand or transfer. Where tried, patients responded to a treatment with pyridostigmine or a combination of pyridostigmine and salbutamol. Patients reported increased power of proximal muscles and increased tolerance to walking and climbing stairs. Neurophysiological abnormalities were consistently found in all cases (Table 1). Of note, not all muscle groups showed neuromuscular junction dysfunction on electromyography. For example, Case 3 had significant decrement (50%) on repetitive nerve stimulation of anconeus muscle but no decrement was detected in abductor digiti minimi (Fig. 2). Similarly, Case 7 had a significant decrement (23–30%) in trapezius muscle, whereas there was no observable decrement when performing repetitive nerve stimulation on abductor digiti minimi. These recordings were consistent with the pattern of muscle weakness observed in the affected individuals. Unusual for CMS, where tested, affected individuals had raised creatine kinase levels.

**Table 1:** Clinical details of cases with *GMPPB* mutations

		Case 1	Case 2	Case 3	Case 4	Case 5	Case 6	Case 7	Case 8	Case 9	Case 10	Case 11
Mutation, DNA		c.79G>C	c.781C>T	c.79G>C	c.308C>T	c.308C>T	c.308C>T	c.79G>C	c.559C>T	c.656T>C	c.656T>C	c.64C>T
		c.859C>T	c.130-3C>G	c.760G>A	homozygous	homozygous	homozygous	c.907C>T	c.578T>C	c.860G>A	c.860G>A	c.1000G>A
Mutation, protein		p.Asp27His	p.Arg261Cys	p.Asp27His	p.Pro103Leu	p.Pro103Leu	p.Pro103Leu	p.Asp27His	p.Gln187^a^	p.Ile219Thr	p.Ile219Thr	p.Pro22Ser
		p.Arg287Trp	Splicing	p.Val254Met				p.Leu303Phe	p.Ile193Thr	p.Arg287Gln	p.Arg287Gln	p.Asp334Asn
Gender		F	F	F	F	F	M	F	F	M	M	F
Age (years)	Presentation	24	15	20	16	22	31	25	1.5	2	2	2.5
	Current	48	68	28	43	45	35	44	17	34	36	14
Presenting symptoms		Limited march tolerance (following influenza)	Unable to run.	Unable to get up from the floor and lift weights.	Difficulty in climbing stairs, cramps.	Difficulty in climbing stairs, cramps.	Difficulty in climbing stairs, cramps.	Unable to climb up ramps.	Episode of generalized sudden weakness.	Global developmental delay.	Global developmental delay.	Seizures.
Ptosis		No	No	No	Mild	No	No	Mild, not fatigable^a^	No	No	No	No
Bulbar weakness		No	No	No	Mild	No	No	No	No	No	No	No
Neck weakness		4+	5	4+	5	5	5	4+	3	4	4	3
Proximal weakness	UL	4	4	3+	4+	4+	5	4	3–4	4−	5	4+
	LL	4−	3+	4	3	3	4+	4	2–3	4 (flex), 3 (ab/ad), 5− (knee)	5	3-4
Distal weakness	UL	5	5	5	5	5	5	5	4+	4+	5	5
	LL	5	5	5	5	5	5	5	4+	4	5	4+
Axial weakness		4	4	5	3	3	5	4+	3	5	5	3
Cognitive delay		No	No	No	No	No	No	No	No	Mild	Moderate	Moderate
RNS												
Muscle, decrement (%)^b^		Anconeus, 42%	Anconeus, 14%	ADM, <10%	APB, <10%			ADM, <10%	EDC, 37%	APB, <10%	APB, <10%	
				Anconeus, 50%	Nasalis, <10%			Trapezius, 23–30%	Trapezius, 25%			
					Trapezius, 20%							
SFEMG		Abnormal	Abnormal	Abnormal	–	–	–	Abnormal	Abnormal	Abnormal (subtle)	Abnormal (subtle)	Normal
Myopathic EMG		Yes	Yes	Yes	Yes	Yes	–	Yes	Yes	Yes	No	–
CK (Normal ≤200)^c^		2800	418	1600	701	–	–	2668	3000	3000	2832	2500
Biopsy	Muscle	Quadriceps		Quadriceps	–	–	–		Quadriceps	Tibialis anterior	–	
	Features	Dystrophic		Dystrophic	–	–	–	Dystrophic	Dystrophic	Dystrophic	–	Dystrophic
	Alpha-DG needs defining in the legend	–		Reduced	–	–	–	Reduced	Reduced	Reduced	–	Reduced
Muscle MRI		–	Abnormal	–	–	–	–	Abnormal	–	–	–	–
Treatment		P, D, S	P, S	P	P	P	P	P, S	–	P	–	–

ADM = abductor digiti minimi; AH = abductor hallucis; APB = abductor pollicis brevis; CK = creatine kinase; D = 3,4-DAP; EDC = extensor digitorum muscle; LL = lower limbs; P = pyridostigmine; RNS = repetitive nerve stimulation; S = salbutamol; UL = upper limbs.

^a^Ptosis appeared at age 26 after developing thyrotoxicosis and receiving treatment for it.

^b^A decrement of >10% in CMAP amplitude indicates a defect in neuromuscular transmission.

^c^The specific reference range for creatine kinase in serum depends on individual laboratories but values >200 are in general considered abnormal.

#### Cases 8–11

Cases 8–11 are previously undescribed cases of GMPPB-MDDG that were compared with CMS Cases 1–7. They had previously been diagnosed with a muscular dystrophy, and underlying mutations in *GMPPB* had separately been identified. Cases 8, 9 and 10 are of Caucasian descent, Case 11 is of Asian descent. Cases 9 and 10 are siblings. There is no known consanguinity in any of the families. Cases 8–11 presented in early childhood (1–2 years of age). Case 8 presented with an episode of generalized sudden weakness, Cases 9 and 10 presented with global developmental delay (although for Case 9 it was initially attributed to a preceding meningitis), and Case 11 presented with seizures. Cases 8, 9 and 11 have weakness in proximal limb muscles, whereas Case 10 has no detectable weakness. Case 8 has fluctuations of muscle strength with infections, and Case 9 reports fatigability, particularly noted when walking uphill. All have elevated creatine kinase levels. Muscle biopsies were obtained from Cases 8, 9 and 11 and showed dystrophic features with reduced α-dystroglycan. Case 9 had a positive response to pyridostigmine as shown by post-treatment muscle strength measures. The time for keeping his arms outstretched increased from 23 to 102 s, and from 12 to 21 s in neck flexion. Of note, from being initially unable to rise up from the floor before treatment, he was able to rise to stand in 5.8 s.

### Identification of mutations in *GMPPB* as a cause of CMS

We performed whole exome sequencing of DNA from Case 1. The resulting data were filtered against the variants listed on 1000 Genome Project, to remove all variants with population frequency of 0.01 or more (Genomes Project *et al.*, 2010). We used ANNOVAR software to annotate and separate non-synonymous substitutions and splicing mutations (Wang *et al.*, 2010). As CMS is usually inherited in an autosomal recessive manner, we focused on all genes that have either one or more homozygous variant, or two or more heterozygous variants in the same gene. We further filtered the obtained variants against an in-house database of 14 exomes from cases with unrelated disorders. This analysis limited the list of possible candidate genes to eight (Supplementary Table 1). The remaining variants were ranked based on their functional annotation and predicted pathogenicity of associated mutations (Supplementary Table 2). The most highly ranked candidate gene resulting from this analysis was *GMPPB*. *GMPPB* encodes GDP-mannose pyrophosphorylase B—an enzyme involved in glycosylation. Mutations in four other glycosylation genes (*ALG2*, *ALG14*, *DPAGT1* and *GFPT1*) are known to lead to the development of CMS, and the symptoms of other glycosylation-CMS patients are similar to the symptoms of Case 1. Therefore, variants in *GMPPB* were good candidates for a potential cause of CMS. We found that Case 1 had two missense mutations in *GMPPB*: p.Asp27His (c.79G > C) and p.Arg287Trp (c.859C>T) (Table 1). Both mutations were confirmed by Sanger sequencing.

The *GMPPB* gene has two isoforms NM_021971 and NM_013334. NM_013334 has eight coding exons and 387 amino acids, whereas NM_021971 has nine coding exons and 360 amino acids. The first seven coding exons of both isoforms are identical. The last eighth exon of NM_013334 is 131 amino acids long. The middle 27 amino acids of the corresponding sequence are spliced out in NM_021971 to produce exon 8 and exon 9. It has previously been shown that isoform NM_021971 is expressed in the skeletal muscle and the brain at much higher levels than isoform NM_013334 (Carss *et al.*, 2013). Additionally, we did not find any variants in the 27 amino acids specific to isoform NM_013334 in any of the cases described below. Therefore, we use RefSeq NM_021971 for the annotation of all variants described in this study.

**Figure 3:**
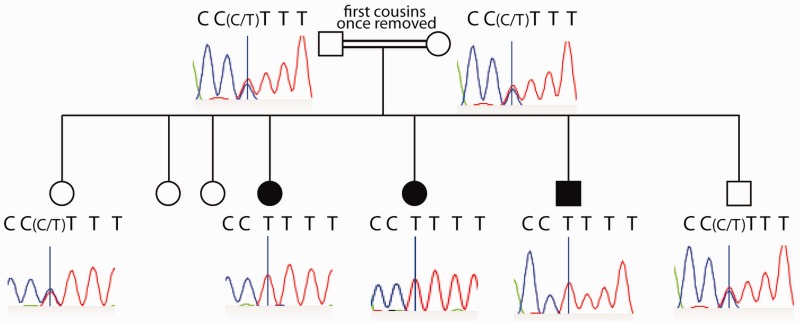
**Co-segregation of CMS phenotype with *GMPPB* mutations in one family (Cases 4–6).** Pedigree symbols are shaded according to the presence of clinical CMS symptoms.

We used Sanger sequencing to screen a cohort of suspected CMS cases for the presence of further possible variants in the *GMPPB* gene. We identified six further cases that had either two heterozygous mutations (Cases 2, 3 and 7) or a homozygous mutation (Cases 4, 5 and 6) in *GMPPB*. Case 2 was compound heterozygote for p.Arg261Cys (c.781C>T) and a potential splicing mutation (c.130-3C>G), Case 3 carried p.Asp27His (c.79G>C) and p.Val254Met (c.760G>A), Case 4 had a homozygous p.Pro103Leu (c.308C>T) mutation, and Case 7 carried p.Asp27His (c.79G>C) and p.Leu303Phe (c.907C>T). Case 4 came from a consanguineous family and had two affected siblings (Cases 5 and 6) and four unaffected siblings. Segregation analysis confirmed that both affected siblings carried a homozygous (c.308C>T) p.Pro103Leu mutation, while two unaffected siblings and both parents carried only one copy of the allele (Fig. 3). Similarly the *GMPPB* mutations segregated with disease on all available family samples from Cases 1, 2, 3 and 7. Thus, we identified seven cases of CMS with mutations in the *GMPPB* gene. Three of the described mutations (p.Asp27His, p.Arg287Trp, p.Arg287Gln) are listed in the Exome Variant Server database ([Exome Variant Server, NHLBI GO Exome Sequencing Project (ESP), Seattle, WA (URL: http://evs.gs.washington.edu/EVS/) (Nov 2014)]). The frequency of the mutations in the general population are 0.077% (p.Asp27His), 0.008% (p.Arg287Trp), and 0.015% (p.Arg287Gln), consistent with the notion of the variants being pathogenic.

Mutations in *GMPPB* can cause muscular dystrophy-dystroglycanopathy [MDDGA14 (MIM 615350), MDDGB14 (MIM 615351), MDDGC14 (MIM 615352)] (Carss *et al.*, 2013; Raphael *et al.*, 2014). The clinical spectrum varies, ranging from a relatively mild phenotype restricted to the limb girdles, through congenital muscular dystrophy with mental retardation, to severe congenital muscular dystrophy with structural brain and eye defects. Serum creatine kinase levels are elevated and analysis of muscle biopsies from the affected individuals showed features characteristic of a muscular dystrophy and reduction in the amount of glycosylated α-dystroglycan. EMG was not performed.

Using Sanger sequencing muscular dystrophy Cases 8–11 were found to have mutations in *GMPPB*. Case 8 has p.Gln187* and p.Ile193Thr, Cases 9 and 10 have p.Ile219Thr and p.Arg287Gln, and Case 11 has p.Pro22Ser and p.Asp334Asn mutations.

Thus, we identify *GMPPB* as a new gene locus in which mutations can underlie a myasthenic syndrome. We also describe four new MDDG patients due to mutations in *GMPPB*.

### Pathogenicity of *GMPPB* mutations

*GMPPB* encodes the enzyme mannose-1-phosphate guanyltransferase beta. It is a cytoplasmic protein that catalyses the formation of GDP-mannose from mannose-1-phosphate and GTP (Ning and Elbein, 2000; Davis *et al.*, 2004). GDP-mannose is used as a building block for multiple glycosylation reactions including *N*- and *O*-linked glycosylation. Knockdown of a *GMPPB* orthologue in zebrafish causes structural muscle and CNS and eye defects, with reduced mobility and reduced glycosylation of α-dystroglycan (Carss *et al.*, 2013).

**Figure 4:**
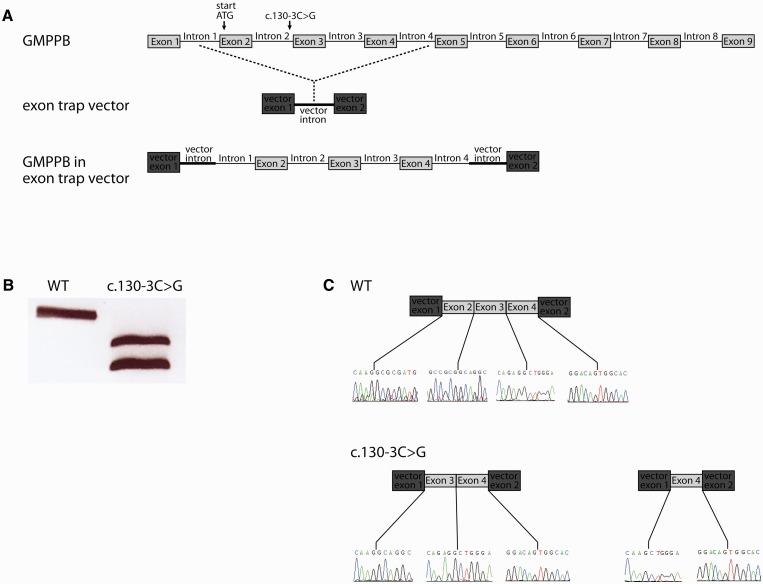
**Mutation c.130-3C>G disrupts wild-type splicing pattern of *GMPPB*.** (**A**) Schematic structure of *GMPPB* gene, the pET01 exon trap vector, and the exon trap vector with inserted *GMPPB* exons 2–4. (**B**) Gel electrophoresis of amplicons generated using vector-specific primers. The wild-type (WT) construct generated one transcript, whereas c.130-3C>G mutant construct generates two shorter transcripts. (**C**) Sequencing data and schematic diagrams showing aberrant splicing from the mutant construct. The nucleotide sequence around each splice site is shown.

To confirm the pathogenicity of the newly identified mutations, we performed a series of *in silico* and expression level studies. Mutation c.130-3C>G alters the nucleotide at the −3 position upstream of 3’ splice site of intron 2 (ENSEMBL transcript ID ENST00000480687). It is located within the pyrimidine tract of the intron within a close proximity of the splice acceptor site. Splice site prediction software Human Splicing Finder v2.4.1 identified that this mutation has the potential to disrupt the wild-type splice site, altering the normal pre-mRNA splicing pattern (Desmet *et al.*, 2009). To test this prediction, we analysed *GMPPB* RNA splicing using exon trapping. The exon trap vector pET01 (MoBiTec) contains two exons separated by an intron sequence containing a multiple cloning site (Fig. 4). We cloned *GMPPB* exons 2, 3, 4 and flanking intronic sequences into the pET01 vector, and introduced the c.130-3C>G mutation by site-directed mutagenesis. Results of the exon trap analysis are shown in Fig. 4. The plasmid carrying wild-type sequence was correctly spliced. The plasmid carrying c.130-3C>G mutation produced two products with different sizes: a larger transcript in which exon 2 was skipped, and a shorter transcript in which both exon 2 and 3 were skipped. The deletion of exon 2 would lead to the loss of the original start codon. The alternative potential translation start site could be either Met56 in exon 3 for the transcript lacking exon 2, or Met77 in exon 4 for the transcript lacking exons 2 and 3. In either case the mutant transcript would lead to the production of a truncated protein.

**Figure 5:**
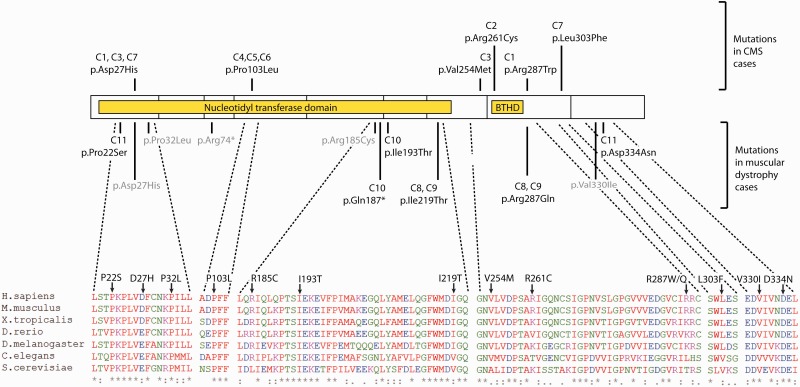
**GMPPB domain structure and conservation.** GMPPB is a 360-aa polypeptide and consists of nine exons (shown with blocks on the scheme). It has two predicted PFAM domains: nucleotidyl transferase domain and bacterial transferase hexapeptide domain (shown with yellow blocks). The scheme shows CMS-associated mutations (above GMPPB scheme), and mutations associated with muscular dystrophy (underneath GMPPB scheme). Mutations described in this paper are shown in black, whereas mutations published previously are shown in grey for comparison. Protein alignment was performed in ClustalW2.

Human GMPPB contains an N-terminal pyrophosphorylase domain harbouring the conserved signature motif for nucleotide binding and transfer, and a putative C-terminal hexapeptide repeat domain expected to form a left-handed beta helix structure (Fig. 5). Of the six CMS-causing missense mutations reported here, two (p.Asp27His, p.Pro103Leu) reside in the N-terminal domain close to the conserved sequence motif, and hence may have a direct impact on catalysis. Both mutations were predicted to be damaging by the CADD algorithm, and one mutation (p.Asp27His) was predicted to be damaging by SIFT algorithm (Ng and Henikoff, 2001; Kircher *et al.*, 2014). The other four mutations (Val254Met, p.Arg261Cys, p.Arg287Trp, p.Leu303Phe) are found in the C-terminal domain and may impact on other non-catalytic properties of the protein (e.g. oligomerization, protein–protein interactions) that remain to be determined. These four missense mutations were predicted to be damaging by CADD and SIFT algorithms, and three of them (Val254Met, p.Arg287Trp, p.Leu303Phe) were predicted to be damaging by PolyPhen-2 software (Adzhubei *et al.*, 2010) (Supplementary Table 3).

**Figure 6:**
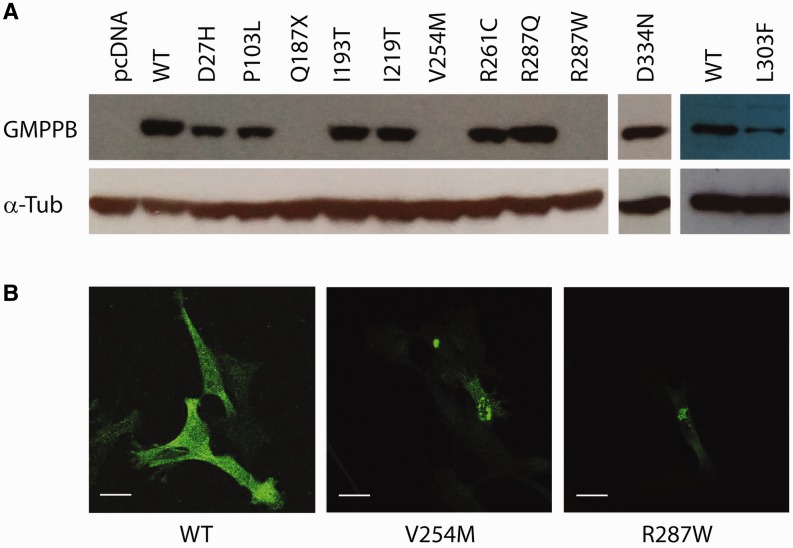
**Effect of different variants on GMPPB expression and localization.** (**A**) GMPPB constructs were transfected into HEK293 cells, protein lysates were prepared 48 h after transfection and analysed by western blot using anti-GMPPB antibody. (**B**) GMPPB constructs were transfected into C2C12 cells. Permeabilized cells were stained with anti-GMPPB antibody. Scale bar = 20 µm.

To determine the effect of the mutations on protein expression, we cloned and expressed wild-type and mutant GMPPB in HEK293 cells. Endogenous levels of GMPPB in HEK293 cells were too low to be detected on the western blot. The construct carrying wild-type GMPPB was well expressed and easily detectable. Two of the newly found mutations caused a drastic reduction of GMPPB expression: no expression was detectable for p.V254M (found in Case 3) and p.R287W (found in Case 1) mutant constructs (Fig. 6A). Additionally, a clear reduction in expression of p.L303F (found in Case 7) was seen.

In addition, we analysed expression and localization of the mutant GMPPB in the mouse muscle C2C12 cell line. In the majority of transfected cells wild-type GMPPB was spread uniformly in the cytoplasm (Fig. 6B). In agreement with the western blot results, expression of p.V254M and p.R287W was drastically reduced, with only few cells expressing detectable level of protein. The cells that did express p.V254M or p.R287W displayed a punctate pattern of GMPPB localization, suggesting that these mutations cause protein aggregation. Thus, some missense mutations in GMPPB affect expression and localization, consistent with the notion of the mutations being pathogenic. Other missense mutations not impairing expression or localization are likely to disrupt GMPPB catalytic activity or interaction with potential partners.

### Neuromuscular transmission defect in patients harbouring *GMPPB* mutations

The GMPPB-CMS cases described above have a clear neurotransmission defect detectable on EMG studies (Table 1 and Fig. 2). To determine whether all *GMPPB* mutations lead to defective neuromuscular transmission and whether the GMPPB-MDDG cases have myasthenic features similar to CMS patients, we performed neurophysiological analysis of GMPPB-MDDG Cases 8–11.

Case 8, with heterozygous mutations in *GMPPB* c.559C>T (p.Q187*) and c.578T>C (p.I193T), showed a clear decrement on repetitive nerve stimulation. Minor defects in neuromuscular junction transmission were also observed in Cases 9 and 10 with an increase in jitter and occasional block on SFEMG, although decrement was not detected. By contrast, in Case 11 there was no evidence from EMG or SFEMG of impaired neuromuscular transmission, and no evidence of fatigable weakness on examination. These results demonstrate that some *GMPPB* mutations may have a clear detrimental effect on neuromuscular transmission (in the case of CMS-associated mutations, and a subset of MDDG-associated mutations), while conversely others may have no detectable effect on signal transmission at the neuromuscular junction (other MDDG patients).

### Myopathic features of GMPPB-CMS

**Figure 7:**
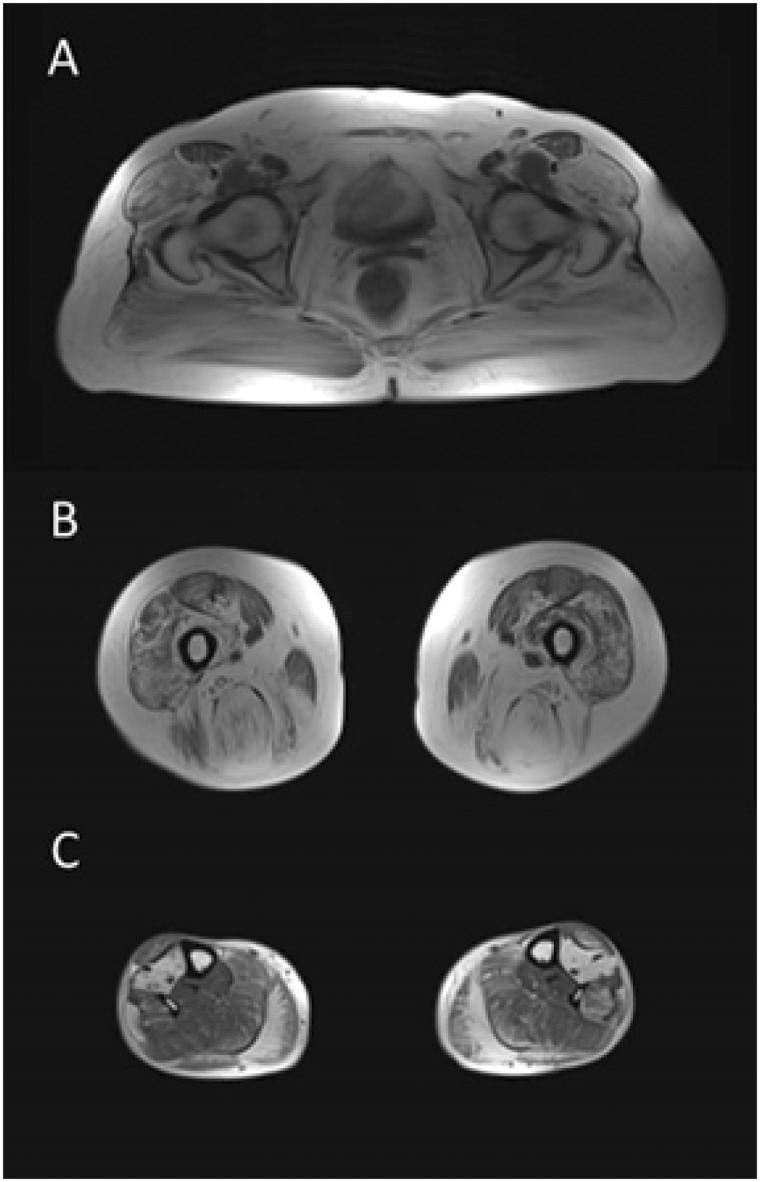
**Muscle MRI from Case 2.** The muscle MRI study (T_1_-weighted sequences) showed consistent abnormalities in gluteal (**A**), thigh (**B**) and calf (**C**) muscles, with confluent areas of increased signal or end-stage appearance but relative sparing of certain muscles, especially at the distal level, in keeping with the clinical and electrophysiological findings.

Whereas a number of the CMS may show some non-specific myopathic features on EMG or on muscle biopsy, myopathic features seem to be an integral part of the GMPPB-CMS. Analysed cases (Cases 1–5 and 7) have definitive myopathic changes on concentric needle electromyography (for example see Fig. 2). All tested cases (Cases 1–4 and 7) have elevated levels of serum creatine kinase indicating muscle damage. Cases 1 and 3 have undergone muscle biopsies and all showed dystrophic features. Case 2 and 7 have undergone a muscle MRI study and both showed selective muscle fibro-fatty replacement, as observed in muscular dystrophies (Fig. 7).

The levels of α-dystroglycan glycosylation in muscle biopsies can be assessed by immunohistochemistry using IIH6 antibody, which specifically recognizes the glycosylated isoform of the protein (Michele *et al.*, 2002). This forms a part of the diagnostic procedure for suspected muscular dystrophy disorders and several GMPPB cases described in this paper underwent such examinations. All analysed cases (Cases 8, 9 and 11 for muscular dystrophy, and Cases 3 and 7 for CMS) had reduced α-dystroglycan staining.

Thus, reduction in α-dystroglycan glycosylation is common to both disorders associated with *GMPPB* mutations, and is likely to underlie the dystrophic features integral to both GMPPB-CMS and GMPPB-MDDG. However, in cases of GMPPB-CMS, it is likely that additional neuromuscular junction proteins are mis-glycosylated, leading to the defective synaptic transmission.

## Discussion

We identify mutations in GMPPB, an enzyme involved in glycosylation, as a new cause of CMS. GMPPB-CMS patients have hallmark myasthenic features—fluctuating fatigable muscle weakness and decrement of compound muscle action potential on repetitive nerve stimulation. The CMS-associated *GMPPB* mutations that we describe are predicted to be pathogenic by several different algorithms, and several cause a distinct reduction in the expressed levels of the protein. Clinically, the GMPPB-CMS cases identified (Cases 1–7) share many of the features that are characteristic for other glycosylation-CMS subtypes (*ALG2-*, *ALG14-*, *DPAGT1*- and *GFPT1*-CMS) (Guergueltcheva *et al.*, 2011; Cossins *et al.*, 2013; Zoltowska *et al.*, 2013). The age of presentation of GMPPB-CMS in adolescence or early adulthood seems later than for most CMS. Muscle weakness is predominantly limited to proximal muscle groups affecting both lower and upper limbs. In common with other CMS due to mutations affecting glycosylation, the facial, eye, and bulbar muscles are largely spared. Ptosis was only present in one of the newly-identified GMPPB-CMS cases, contrasting with many other CMS subtypes where ptosis often serves as an important clinical clue for considering CMS. As in other glycosylation-pathway CMS, affected individuals report a beneficial response to pyridostigmine or pyridostigmine plus salbutamol.

*GMPPB*-associated muscular dystrophy cases show a reduction in the glycosylation level of α-dystroglycan (Carss *et al.*, 2013). α-Dystroglycan is an extracellular protein that is non-covalently linked to the transmembrane β-dystroglycan (Holt *et al.*, 2000). α-Dystroglycan interacts with several extracellular matrix components, while β-dystroglycan interacts with intracellular cytoskeleton. Thus, the dystroglycan complex provides a link between the extracellular matrix and intracellular machinery. Under normal conditions, α-dystroglycan is heavily *O*-glycosylated and this glycosylation is essential for efficient interaction with its extracellular partners (Michele *et al.*, 2002; Michele and Campbell, 2003). Reduction in the glycosylation level disrupts these molecular interactions and contributes to destabilization of the sarcolemma, contributing to muscle damage during contraction. GMPPB-MDDG patients have a set of characteristic dystrophic features and a variable degree of structural brain and eye abnormalities. At the severe end of the spectrum, mutations in *GMPPB* lead to the congenital muscular dystrophy with brain and eye abnormalities. At the mild end of the spectrum, mutations in *GMPPB* lead to the limb-girdle muscular dystrophy that is limited to a weakness in the proximal limb muscles.

Similar to GMPPB-MDDG, GMPPB-CMS have a set of myopathic features that are detectable on muscle biopsies, muscle MRI, concentric needle EMG, and through elevated serum creatine kinase levels. Although several CMS subtypes such as slow-channel syndrome (Chaouch *et al.*, 2012), DOK7-CMS (Müller *et al.*, 2007) and GFPT1-CMS (Guergueltcheva *et al.*, 2011) can have mildly raised creatine kinase values, this is rare (apart from in GFPT1-CMS, not seen in over 300 CMS cases analysed in Oxford), and when reported has rarely been more than two to three times normal values. All the GMPPB-CMS patients have a characteristic neuromuscular transmission defect that is detectable by repetitive nerve stimulation EMG studies on the affected group of muscles. A subset of GMPPB-MDDG patients display a neuromuscular transmission defect, whereas other GMPPB-MDDG patients do not. Thus mutations in *GMPPB* provide a link between myasthenic syndromes and dystroglycanopathies. A relationship between CMS and dystroglycanopathies has previously been proposed (Compton *et al.*, 2008); however, this was for a lethal form of congenital myopathy due to mutations in *CNTN1* where affected individuals died at birth or shortly afterwards. The wide spectrum of clinical features with *GMPPB* mutations is likely due to its ubiquitous expression, and its involvement in the glycosylation of different proteins. It is not clear at present why different mutations in the same gene can lead to the different clinical manifestations.

In summary, we identify mutations in *GMPPB* as a novel genetic cause of impaired signal transmission at the neuromuscular junction. We find that mutations in *GMPPB* can lead to overlapping phenotypes with a spectrum of different clinical outcomes. At one end of the spectrum are cases in which fatigable weakness with a characteristic neuromuscular junction transmission defect, is the major symptom, at the other end mutations in *GMPPB* lead to the onset of muscular dystrophy with no demonstrable effect on the neuromuscular junction. CMS due to *GMPPB* mutations may frequently remain undiagnosed due to lack of facial features usually associated with myasthenia, the presence of high creatine kinase levels and the restricted muscle groups that show decrement on repetitive nerve stimulation. Recognition of this condition is important as these patients respond symptomatically to appropriate medication and their quality of life can be significantly improved by appropriate treatment.

## Web resources

ClustalW2, http://www.ebi.ac.uk/Tools/msa/clustalw2/

Exome Variant Server, http://evs.gs.washington.edu/EVS/

OMIM, http://www.omim.org/

## Abbreviations

CMS: congenital myasthenic syndromes
MDDG: muscular dystrophy dystroglycanopathy
